# Factors influencing perceived benefits and behavioral intention to use mental health chatbots among professional employees: an empirical study

**DOI:** 10.3389/fdgth.2025.1606273

**Published:** 2025-11-11

**Authors:** Gehad Mohammed Ahmed Naji, Foo Yuan, Nurul Azzura, Fajer Danish, Ali Ateeq, Siddig Balal Ibrahim, Halimaton Hakimi, Aziah Binti Abdollah, Yulita Hanum P Iskandar

**Affiliations:** 1 Graduate School of Business, Universiti Sains Malaysia (USM), Pulau Pinang, Malaysia; 2 Royal University for Women, Sanad, Bahrain; 3 Administrative Science Department, College of Administrative and Financial Science, Gulf University, Sanad, Bahrain; 4 Positive Computing Research Centre, Universiti Teknologi, Perak, Malaysia; 5 Asia Pacific University of Technology & Innovation (APU), Kuala Lumpur, Malaysia

**Keywords:** mental health chatbots, professional employees, Malaysia, perceived benefits, attitude, technology acceptance, health system access

## Abstract

**Purpose:**

This study explores factors influencing Malaysian professionals' intentions to use mental health chatbots by integrating the Unified Theory of Acceptance and Use of Technology (UTAUT) and the Theory of Planned Behaviour (TPB). It examines UTAUT factors' direct effects on usage intention and the mediating role of perceived benefits, along with the moderating influence of attitudes towards chatbots.

**Research design & methodology:**

The study collects data from 351 professional employees in Malaysia using an online survey and analyses it using structural equation modelling (SEM).

**Findings:**

The study outcomes indicate that UTAUT factors significantly influence perceived benefits (*β* = 0.793, *p* < 0.001; *R*^2^ = 0.601). However, perceived benefits did not significantly predict behavioural intention (*β* = 0.107, *p* = 0.464; *R*^2^ = 0.449). Attitudes towards chatbots showed only a weak moderating effect on the UTAUT–perceived benefit relationship (*β* = 0.009, *p* = 0.094), while other hypothesised moderating effects were not supported. These findings suggest a more complex interplay of factors influencing the adoption of mental health chatbots in professional settings than previously assumed.

**Conclusion:**

These findings challenge the assumption that perceived benefits alone drive adoption. They suggest a more complex interplay of factors influencing behavioural intention, indicating that trust, privacy, and credibility may play more critical roles in shaping adoption decisions.

**Implications:**

The study provides valuable insights for developers and implementers of mental health technologies. While UTAUT factors are crucial in shaping perceived benefits, the lack of a direct link to behavioural intention highlights the need to explore additional psychological and contextual factors. Future research should consider longitudinal designs and probabilistic sampling to enhance generalisability and causal inference.

## Introduction

**Figure 1:**
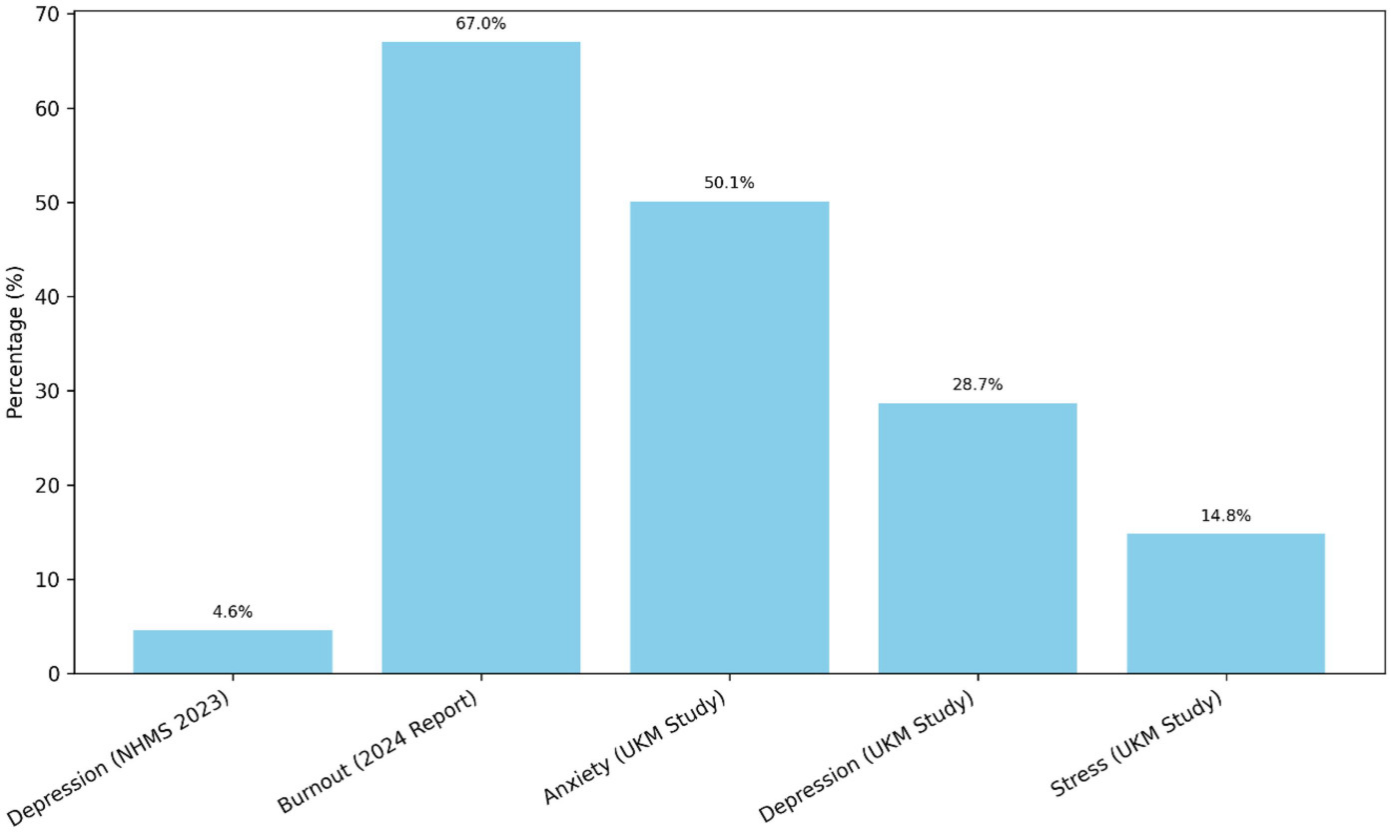
Prevalence of mental health issues among Malaysian professionals.

A person's mental health has profound consequences for other aspects of their lives, including their physical health, fulfilment, relationships, accomplishment at work, and overall quality of life (1). But mental health issues can be prevalent and debilitating for people everywhere, especially among professionals who struggle with a wide range of stresses and pressures on the job. Professionals include managers, physicians, engineers, educators, auditors, and others who have extensive education and expertise in the fields of their choice. These employees frequently face intense stress due to the many demands placed upon them (2). A lack of autonomy, lack of support, lack of recognition, and a lack of work–life balance are also possible concerns. Stress, anxiety, depression, substance abuse, and bipolar disorder are just some of the mental health issues that can be triggered or aggravated worse by the environment in which many professionals work (3). In Malaysia, mental health concerns among working adults have reached alarming levels. According to the National Health and Morbidity Survey (NHMS) 2023, approximately one million Malaysians aged 15 and above suffer from depression, with the number doubling since 2019 (4). A 2024 Wellness at Work Report revealed that 67% of Malaysian employees are experiencing burnout, with millennials being the most affected group (69%) (5). Furthermore, a study conducted among staff at Universiti Kebangsaan Malaysia found that 50.1% experienced anxiety, 28.7% depression, and 14.8% stress symptoms (6). Figure 1 highlights the urgent need to address mental health challenges in professional settings and explore innovative support mechanisms such as mental health chatbots (6).

Professionals, their employers, and society are all vulnerable to the negative effects of mental health issues (7). A person's ability to think clearly, make sound judgements, be creative, work efficiently, and produce high-quality work can all be negatively impacted by mental health issues. They can additionally increase the likelihood of employee loss of talent, presenteeism (going to work despite being sick), dissatisfaction, and conflict at work. Furthermore, professional employees' mental health issues can have adverse effects on their loved ones and the communities in which they live through things such as emotional distress, marital difficulties, isolation, and stigma. Therefore, it is essential to care for the mental health of professionals by providing them with effortlessly accessible and effective treatments (8, 9).

One of the latest innovations for mental health is chatbot technology. In 2021, a team from Monash University Malaysia, led by Professor Dr. Su Tin Tin at the Jeffrey Cheah School of Medicine and Health Sciences, developed a chatbot designed to provide cognitive behavioural therapy (CBT) to nurses experiencing occupational burnout (10). This innovation, as reported by [Malay Mail 2021; Bernama (108)]., aimed to address the challenges of mental health stigma and resource scarcity in Malaysia by offering a fully automated conversational agent for support. Chatbots are software programmes that can interact with a user through text or voice. Users can access chatbots through various platforms, such as websites, mobile apps, messaging services, social media networks, and voice assistants (11, 12). Chatbots can enhance their conversational skills and offer more personalised responses by using artificial intelligence (AI) techniques, such as machine learning (ML), natural language generation (NLG), and natural language understanding (NLU) (109). Chatbots can deliver a range of mental health services, such as psychoeducation, self-help, screening, feedback, counselling, coaching, peer support, and referral. A report by Grand View Research (2019) estimated that the global market value of mental health chatbots was USD 314.3 million in 2019 and predicted that it would grow at a compound annual growth rate (CAGR) of 26.4% from 2020 to 2027 (13, 14). The main factors driving this market growth are the growing need for mental healthcare services, especially during the COVID-19 pandemic, and the increasing adoption of digital technologies for mental health. However, there is a gap in the research on how different user segments view and use mental health chatbots, especially among professional employees who encounter various stressors and challenges at work (15).

Globally, the chatbot market for mental health and therapy is poised for significant growth, driven by technological advancements in AI and a burgeoning awareness of mental health needs. Precedence Research (2023) mentioned that the market revenue is projected to reach approximately USD 3.390 million by 2029, with advancements in natural language processing (NLP) and machine learning (ML) algorithms enhancing the capabilities of these chatbots (16). The demand for chatbots in this sector is partly driven by the shortage of mental health professionals worldwide, with the WHO reporting only 13 mental health professionals for every 100,000 persons, significantly fewer in low-income nations (110). In the context of Malaysia and similar regions, the integration of chatbots in mental health services addresses both the stigma surrounding mental health and the scarcity of resources. Technologies such as machine learning and deep learning dominate the chatbot market, offering potential for personalised therapy and support. North America currently holds the largest market share, attributed to a high prevalence of mental health disorders and a strong focus on digital health solutions (17, 18). However, the Asia Pacific region, including countries such as Malaysia, is expected to witness the fastest growth due to rapid urbanisation, changing lifestyles, and increasing mental health awareness.

Patients seeking mental health consultation traditionally rely on face-to-face therapy sessions, which can be limited by availability, cost, and the stigma associated with seeking help. Chatbots offer an alternative by providing accessible, immediate, and stigma-free support. They're available through various platforms, including websites, apps, and social media, making them a versatile tool for mental health support. The level of acceptance among mental health patients for using chatbots varies. Some appreciate the anonymity and convenience, especially for initial screenings or when seeking general advice. However, others may prefer human interaction, especially for more complex or sensitive issues, due to the perceived lack of empathy and understanding from chatbots. The effectiveness and acceptance of mental health chatbots hinge on their ability to provide empathetic, accurate, and personalised support. Continuous improvements in AI and careful design to address the nuances of mental healthcare are essential for these tools to be effectively integrated into the broader mental health support (19–21).

There may be several benefits for professionals using chatbots for mental health over more conventional methods of mental healthcare. When compared with traditional face-to-face or online therapy sessions, chatbots may offer several advantages, including being more accessible, scalable, cost-effective, confidential, and individualised. Chatbots have the potential to remove barriers such as prejudice, geographic separation from a therapist, cost, and lack of accessibility that prevent professionals from accessing mental health services (10, 22). As a result of having access to information, support, and self-management tools, chatbots may also enable professional employees to actively participate in their own mental health treatment and recovery. Malaysian workers are exhausted and sleepless, with more than half of them experiencing at least one aspect of work-related stress and getting less than 7 h of sleep in a day (23). The report also indicated that mental health issues are increasing among Malaysian employees, with 22% of them having financial worries and 20% of them facing workplace harassment. Another report (111) revealed that the occurrence of mental health issues in Malaysia as of June 2019 differed by demography. The report found that 11% of respondents aged 18–24 years old reported having mental health issues, compared with 2% of those aged 55 years old and above. Respondents who earned >7,000 ringgits were more likely to have mental health issues, with 13%, than those who earned <3,000 ringgits, with 6%. Therefore, it is essential to address the needs of professional employees' mental health by offering them effective and feasible interventions (24).

However, there are limitations and difficulties associated with the use of chatbots to provide mental healthcare. One difficulty is that AI currently lacks the capacity to recognise and appropriately respond to the wide range of human emotions and experiences (25). Problems could arise in dealing with serious mental health crises, emergencies, or situations that require human intervention if this is the case. Problems with informed consent, data security, quality control, and professional standards also arise from ethical, legal, and privacy issues. User distrust, apathy, or dissatisfaction due to technical issues or a preference for human interaction is a third obstacle. Moreover, there is a lack of research and evaluation into the effectiveness, usability, and acceptability of chatbots in various mental health configurations (26).

As a result, it is crucial to learn what factors influence professional employees' behavioural intention (BI) to use chatbots for mental health. The term “behavioural intention” refers to a person's intended use for utilising certain types of technology (27). Individual characteristics (such as gender, age, and experience), psychological factors (such as attitude, subjective norm, and perceived behavioural control), and technological factors (such as performance expectancy, effort expectancy, social influence, and facilitating conditions) all play a role in shaping behavioural intentions. When these considerations are taken into account, it becomes possible to create mental health chatbots that are more likely to be adopted by the professional workforce (28).

While previous research has explored general users' acceptance of health technologies, there remains a limited understanding of how professional employees perceive and adopt mental health chatbots. This group represents a distinct demographic, highly educated, technologically proficient, and often subject to considerable workplace stress (29, 30). Gaining insight into their behavioural intentions is crucial for designing interventions that are both effective and user-friendly.

This study combines the Unified Theory of Acceptance and Use of Technology (UTAUT) with the Theory of Planned Behaviour (TPB) to investigate Malaysian professionals' willingness to engage with mental health chatbots. It examines key UTAUT constructs, i.e., performance expectancy, effort expectancy, social influence, and facilitating conditions, while also exploring the mediating role of perceived benefits (PB) and the moderating effect of attitudes towards chatbots. To the best of our knowledge, this is among the first studies in Malaysia, and one of the few globally, to analyse these dynamics within a professional workforce context.

## Literature review and hypothesis development

### An overview of mental health issues

One in four people may have mental health disorders at some point in their lives, making them noticeable and burdensome for people all over the world. One of the primary contributors to disability and death worldwide is mental illness. The most prevalent mental health conditions include substance abuse, bipolar disorder, stress, anxiety, and depression (31).

Physical health, psychological well-being, social connections, productivity at work, and quality of life are just a few of the functioning aspects that can be impacted by mental health problems (32). For instance, depression can result in low mood, loss of interest, exhaustion, insomnia, changes in appetite, and suicidal thoughts. Anxiety is associated with excessive worry, fear, tension, panic attacks, and rejection of specific situations. Stress-related symptoms include headaches, muscle tension, irritability, insomnia, and hypertension (33). These mental health issues also raise the risk of developing other chronic conditions such as dementia, diabetes, obesity, cancer, and cardiovascular diseases. Stress-related symptoms include headaches, muscle tension, irritability, insomnia, and hypertension. These mental health issues also raise the risk of developing other chronic ailments such as dementia, diabetes, obesity, cancer, and cardiovascular diseases (34).

Due to different work-related circumstances, professional employees comprise a particular population that is vulnerable to mental health issues (35). Professional employees are people with specialised knowledge and abilities in a certain industry or profession, including managers, doctors, engineers, teachers, accountants, and others. These workers frequently work under intense pressure from rival employees, responsibilities, workload, deadlines, and expectations. Together with these issues, they might also encounter role conflict, role ambiguity, role overload, lack of autonomy, a lack of support, a lack of acknowledgment, and a lack of work–life balance (36). These elements may cause the beginning or aggravation of mental health problems in professional employees.

Professional employees who experience mental health problems may suffer adverse effects on themselves, their employers, and society. For instance, mental health problems may impair one's cognitive function, ability to make decisions, creativity, productivity, and output quality (37). Moreover, they can raise the likelihood of turnover, presenteeism (working while sick), employee dissatisfaction, and workplace issues. Furthermore, professional employees' issues with mental health can have an impact on their families, friends, and communities by leading to emotional distress, marriage challenges, social isolation, and stigma. Therefore, it is critical to address the mental health concerns that they face to assist professional employees, deal with their psychiatric conditions, and improve their well-being. This may be done by offering them effective and accessible therapies.

### Technological trends in addressing mental health issues

A growing variety of aspects of mental healthcare, including assessment, diagnosis, monitoring, intervention, evaluation, and communication, are supported by technology. Technology-based interventions can be more convenient, scalable, cost-effective, confidential, and personalised than traditional forms of mental health therapy. Technology can also help people who face difficulties such as stigma, distance from a therapist, financial limitations, and time constraints to seek or receive mental healthcare (38).

Some examples of technology-based interventions for mental health include online platforms, mobile applications, web-based programmes, computerised cognitive behavioural therapy (cCBT), telepsychiatry, virtual reality (VR), gaming, wearable devices, and chatbots. Services such as psychoeducation, self-help, screening, feedback, therapy, coaching, peer support, and referral are just a few of the many services that these treatments can offer (30, 39). Also, they can focus on a variety of groups, including minorities, refugees, adults over the age of 18, older adults, and those with particular needs or preferences (40). The platform technology that is used to offer services may be utilised to categorise technology-based therapies for mental health. A recent scoping assessment identified three categories of digital mental health therapies based on this criterion: web-based, mobile-based, and external device-based interventions. Web-based interventions are those that can be accessed via a computer or laptop and a website or web-based programme (41, 42). Mobile-based interventions can be accessed via a tablet or smartphone and a messaging app (43). External device-based treatments involve interacting with the user through a different device, such as a wearable sensor, a virtual reality headset, or a gaming console. These types of interventions can vary in their functions, features, and formats, depending on the target population, the mental health problem, and the intervention goal. Some examples of technology-based interventions for mental health are listed as follows.

Technology-based interventions have become increasingly prevalent in mental healthcare, offering accessible and scalable solutions (44). These interventions can be categorised into three main types: web-based, mobile-based, and external device-based interventions.

Web-based interventions utilise online platforms to provide mental health support, including psychoeducation, self-help tools, screening, feedback, therapy, coaching, peer support, and referral services (41). These platforms offer a structured approach to mental healthcare, making professional resources more accessible to users. Some notable examples include Togetherall, ReachOut, and UCLA STAND, which provide users with valuable support networks and self-guided interventions (45).

Mobile-based interventions offer similar functionalities but with greater convenience and accessibility through smartphone applications. These apps allow users to track their mood, engage in guided meditation, access therapy chats, and receive mental health support anytime and anywhere. Popular mobile-based interventions include Wysa, 7 Cups, and Happify Health, which integrate AI-driven chatbots and community-based support to enhance user experience and engagement (46).

External device-based interventions involve wearable or interactive technologies that monitor physiological and behavioural data, such as mood fluctuations, activity levels, sleep patterns, and stress indicators. Additionally, some interventions use virtual reality (VR) and gamification to create immersive environments that support mental health treatment (47). Examples include Fitbit, Muse, and Empatica E4, which are wearable monitoring devices, while VR-based interventions such as Bravemind, Limbix Spark, and Psious provide immersive therapeutic experiences. Gamified therapy tools, including SPARX, SuperBetter, and MindLight, leverage game mechanics to improve mental well-being and encourage user engagement (48).

Despite their benefits, technology-based interventions pose several challenges and ethical concerns. Issues related to data security, informed consent, and confidentiality must be carefully addressed to ensure user trust and compliance with ethical guidelines (49). Additionally, maintaining quality control and clinical efficacy remains a critical challenge, as many digital mental health solutions lack rigorous validation and oversight. Another major concern is user engagement, as the effectiveness of these interventions depends on their accessibility, ease of use, and the ability to sustain long-term participation.

Beyond individual users, technology has the potential to transform mental health research and clinical practice. It enables real-time data collection from diverse sources, such as social media, digital pills, and symptom trackers, helping clinicians make data-driven decisions and allocate resources more effectively. By providing real-time feedback on mental health needs and treatment outcomes, technology enhances personalised care and intervention strategies (50, 51). Additionally, it empowers individuals to take an active role in their mental health by granting access to educational resources, self-management tools, and virtual support communities.

To maximise the benefits of technology-based interventions, it is crucial to adopt a multidisciplinary and participatory approach. Collaboration among healthcare professionals, researchers, policymakers, and end-users is essential to developing interventions that are not only effective but also culturally sensitive, ethically sound, and widely accessible (52). Addressing ethical, legal, and regulatory challenges will be key to ensuring the successful integration of technology into mental healthcare.

### Mental health chatbot

A mental health chatbot, which is a computer programme that replicates human communication through text or speech, is one of the revolutionary types of technology-based interventions for mental health. Users can communicate with chatbots through a variety of channels, including websites, smartphone applications, messaging services, social media, and voice assistants. In hopes of enhancing their conversational skills and delivering more customised and adaptive responses, chatbots can also leverage AI techniques such as machine learning (ML), natural language generation (NLG), and natural language understanding (NLU) (53). The use of chatbots has spread to several industries, including customer service, education, entertainment, and healthcare (54). In the context of mental health treatment, chatbots can offer a variety of services, including psychoeducation, self-help, screening, feedback, counselling, coaching, peer support, and referral (55).

Mental health chatbots are a form of AI that is aimed at assisting users with their mental health. These are online services that can be accessed via websites or mobile applications for a modest monthly fee (10). Mental health chatbots can perform a variety of functions, such as having a conversation with the user about their mental health, providing instant 24/7 available chat, delivering detached statistics for the user to self-regulate their mental state, and providing self-assessment and guidelines to help users overcome depression, stress, anxiety, sleep issues, and other conditions (56).

In recent years, chatbots have grown in popularity, particularly among young folks who are more accustomed to technology and more at ease communicating online. Yet, there is a deficit of studies on how professional employees, who could experience various stressors and difficulties at work, use mental health chatbots (57). Professional employees are individuals who undertake difficult jobs with a high degree of autonomy and responsibility. They have specialised knowledge and abilities in their industries. Doctors, attorneys, engineers, teachers, and other professionals are a few examples of professional employees (58).

In order to meet their needs for efficient, affordable, and confidential mental health help, professional employees may find it advantageous to use mental health chatbots. However, there may also be some obstacles or difficulties that hinder users from using mental health chatbots, such as a lack of confidence, privacy concerns, stigma, or a preference for human engagement (59). Mental health chatbot also encounters some challenges in mental healthcare. One challenge is the limitation of AI in fully comprehending and responding to the complex and diverse emotions and experiences of human users, which causes difficulty in handling emergencies, crises, or severe mental health issues that require human intervention or referral (59). Moreover, there are ethical, legal, and privacy concerns related to informed consent, data security, quality control, and professional standards. Furthermore, there is user resistance, distrust, or dissatisfaction due to technical glitches, lack of empathy, or preference for human interaction. Additionally, there is limited evidence or evaluation on the clinical efficacy, usability, and acceptability of mental health chatbots for different groups and contexts. Therefore, it is important to understand the factors that influence their behavioural intention to use mental health chatbots, which is defined as the degree to which a person plans or intends to use a certain technology in the future.

### Theoretical background

This research uses the Unified Theory of Acceptance and Use of Technology (UTAUT) and the Theory of Planned Behaviour (TPB) as the main frameworks to examine how users' intention to adopt and use mental health chatbots is shaped by personal, psychological, and technological factors (60). The UTAUT model consists of four main factors: social influence, performance expectancy, effort expectancy, and facilitating conditions. The TPB model includes three main factors: perceived behavioural control, subjective norm, and attitude. These models have been adapted and expanded to various situations and goals of using mental health chatbots by adding other factors, such as perceived benefits and attitude towards chatbots (26). This research also investigates how user characteristics, such as gender, age, experience, and usage, affect the links between different factors and intention. The following sections will review the existing literature on these models and variables, and their relevance and suitability for this research context.

### Unified Theory of Acceptance and Use of Technology (UTAUT)

UTAUT is a model that explains how users choose to adopt and use an information system. Venkatesh developed this theory by combining and consolidating eight prior models that had investigated information systems usage behaviour (61). The model suggests that user intentions and behaviour are influenced by four main factors: performance expectancy, effort expectancy, social influence, and facilitating conditions. The first three factors have a direct impact on user intentions and behaviour, while the fourth factor only affects user behaviour. The model also indicates that user characteristics, such as gender, age, experience, and voluntariness of use, can alter the effects of the four main factors on user intentions and behaviour. UTAUT has been used to study the acceptance and use of technology in various contexts and domains, such as mobile services, social media, open government data, and health IT (62). UTAUT has also been expanded and revised by later researchers to include additional constructs or variables, such as hedonic motivation, price value, habit, personal innovativeness, trust, anxiety, and self-efficacy. UTAUT has been shown to account for 70% of the variation in behavioural intention to use (BI) and approximately 50% in actual use, which makes it one of the most powerful and comprehensive models in the technology acceptance literature (63).

UTAUT is a widely applied and tested framework in various domains and settings, such as e-government, healthcare, mobile banking, enterprise systems, and mobile internet. However, UTAUT also encountered some challenges and criticisms, such as its failure to account for behavioural intention in diverse scenarios and its restricted generalisability, inflated citations, and methodological problems with the creation and validation of the measures (64). Therefore, some scholars suggested extensions or adaptations of UTAUT to address these issues and enhance its explanatory power. One of the most notable extensions of UTAUT is UTAUT2, to investigate the adoption and use of technology in a consumer setting (65). UTAUT2 introduced three new factors to the original UTAUT model: hedonic motivation, price value, and habit. These factors were found to be important influences on consumers' intention to use or actual use of technology. UTAUT2 also eliminated the voluntariness of use moderator and changed some relationships among the factors to suit the model to the consumer technology use setting. UTAUT2 was found to explain 74% of the variation in behavioural intention and 52% of the variation in technology use. Another adaptation of UTAUT is UTAUT3, to address some theoretical and methodological shortcomings of UTAUT (66). UTAUT3 added attitude as a partial mediator of the effects of exogenous factors on behavioural intention. UTAUT3 also modified some relationships among the factors and moderators based on a meta-analysis and structural equation modelling (SEM) of previous studies that used UTAUT. UTAUT3 was found to explain 75% of the variation in behavioural intention and 53% of the variation in technology use. These adaptations of UTAUT show the strength and adaptability of the framework to fit different settings, domains, technologies, and user groups. They also show the ongoing development and improvement of the theory based on empirical evidence and theoretical arguments.

### Theory of Planned Behaviour (TPB)

According to the TPB framework, three types of beliefs influence human behaviour: behavioural, normative, and control (67). Behavioural beliefs refer to the expected consequences and values of the behaviour. Normative beliefs relate to the social norms and pressures that affect the behaviour. Control beliefs involve the perceived ease or difficulty of performing the behaviour and the factors that can help or hinder it. These three kinds of beliefs influence attitude towards the behaviour, subjective norm, and perceived behavioural control, respectively. These three variables then determine behavioural intention, which is assumed to be an immediate antecedent of behaviour (68). The TPB framework has been widely used and validated in various contexts and domains, such as health, education, environment, and technology. However, TPB also faced some limitations and critiques, such as its neglect of affective and moral factors, ambiguity in defining and measuring subjective norm and perceived behavioural control, assumption of rationality and consistency in human behaviour, and lack of attention to post-intentional factors (69). Therefore, some researchers proposed extensions or modifications of TPB to address these gaps and improve its explanatory power.

One of the most prominent extensions of TPB is TPB with descriptive norms (TPB-DN), to incorporate descriptive norms into TPB (70). Descriptive norms are the beliefs about what most people do or how they behave in a given situation. They are different from injunctive norms, which are the beliefs about what most people approve or disapprove of. Roland et al. (71) argued that descriptive norms can influence behavioural intention and behaviour independently of injunctive norms and other TPB variables (71). They tested their model in four studies involving different behaviours (i.e., binge drinking, blood donation, exercise, and healthy eating) and found that descriptive norms significantly predicted behavioural intention and behaviour over and above injunctive norms. Another extension of TPB is TPB with moral norms (TPB-MN), which was proposed by Liu et al. (72) to include moral norms into TPB. Moral norms are the personal feelings of moral obligation or responsibility to perform or not perform a certain behaviour (72). They are different from social norms, which are the perceived expectations of others or society. Moral norms can influence behavioural intention and behaviour independently of social norms and other TPB variables. They tested their model in two studies involving different behaviours (i.e., cheating on an exam and recycling) and found that moral norms significantly predicted behavioural intention and behaviour over and above social norms (73). By incorporating descriptive norms and moral norms into TPB, these extensions illustrate the various and intricate aspects of human behaviour in particular situations and fields. They also reflect the ongoing progress and improvement of the theory based on empirical data and logical reasoning.

### UTAUT and TPB integration framework

The Unified Theory of Acceptance and Use of Technology (UTAUT) is a framework that integrates some aspects from previous frameworks of technology acceptance, such as the technology acceptance model (TAM) and the theory of reasoned action (TRA). The UTAUT framework suggests that four main factors influence the intention and behaviour of users to adopt and use a technology: performance expectancy, effort expectancy, social influence, and facilitating conditions (74). These factors are also affected by the user's personal characteristics, such as gender, age, experience, and voluntariness of use (81). Osta et al. (75) applied the UTAUT framework to examine the acceptance of AI conversational agents (chatbots) in online health communities (OHCs). They evaluated how the UTAUT factors (performance expectancy, effort expectancy, social influence, and facilitating conditions) influenced the intention and behaviour of OHC users towards chatbots. They discovered that chatbots can enhance the interaction and cooperation among online health communities (OHCs) members and provide personalised health information and support.

The TPB is a psychological theory that links beliefs to behaviour. The theory maintains that three core components, namely, attitude, subjective norms, and perceived behavioural control, together shape an individual's behavioural intentions (76). Attitude is the person's positive or negative appraisal of the behaviour. Subjective norms are the perceived social pressure or encouragement for the behaviour or its avoidance. Perceived behavioural control is the perceived ease or difficulty of performing the behaviour and the availability of resources and opportunities (77). Grové and others created a chatbot for mental health and well-being with and for young people using a participatory design method (15). They used the TPB as a theoretical foundation to develop their research questions and analyse their data. They found that attitude, subjective norms, and perceived behavioural control were important factors influencing young people's intention to use the chatbot. They also found that young people liked the chatbot's personality, character design, conversation style, and content.

Both models have been applied and adapted to study users' intention to use VR for various contexts and purposes. The concepts of UTAUT and TPB were used to investigate users' VR tourism behaviour intention (78). They added perceived benefits as a mediator between UTAUT and behavioural intention, and attitude as a moderator between UTAUT, perceived benefits, and behavioural intention. They found that UTAUT indirectly influenced behaviour through perceived benefits, and attitude positively strengthened these relationships.

These studies show how the UTAUT and TPB are viable models for comprehending and forecasting the behavioural intention to utilise mental health chatbots across a range of settings and groups. However, they also propose that other variables or moderators that are specific to apply on this research on chatbots for mental health should be considered and further researched.

### Variables for UTAUT and TPB integration framework

This study proposes a combined framework that merges the variables of UTAUT and TPB to explore the factors that affect users' intention and behaviour to use a mental health chatbot. UTAUT and TPB are two of the most prevalent and reliable models in the technology acceptance literature. They have some common constructs, such as attitude, perceived benefits, and behavioural intention, but also have some distinct constructs, such as performance expectancy, effort expectancy, social influence, and facilitating conditions in UTAUT. This study aims to offer a more complete and solid explanation of users' acceptance and use of technology by merging these two models. This section will examine the literature on each variable of the UTAUT and TPB combined framework and formulate the hypotheses based on the theoretical and empirical evidence.

### Performance expectancy

Performance expectancy is one of the main factors of UTAUT that explains users' intention to use a technology. It is described as “the extent to which an individual believes that using the system will help him or her to achieve improvements in job performance” (79). It is related to other factors in previous models, such as perceived usefulness, extrinsic motivation, job fit, relative advantage, and outcome expectations (79). Performance expectancy represents the practical and functional value of using a technology for users. Performance expectancy is also affected by several factors, such as gender, age, experience, and voluntariness of use. For instance, performance expectancy had a stronger impact on behavioural intention for men than for women and for younger users than for older users (79). They also discovered that performance expectancy was more relevant in mandatory settings than in voluntary settings. Performance expectancy is a crucial factor to understand users' acceptance and use of technology because it captures the perceived advantages and outcomes of using a technology. Users are more inclined to adopt a technology if they believe that it will improve their performance, productivity, or efficiency. Therefore, it is vital to design and implement technologies that can satisfy users' expectations and needs in terms of performance.

### Effort expectancy

One of the main factors of the UTAUT model, which studies how users accept and use technology based on their expectations of how well it works, how easy it is, how others view it, and how it fits their needs, is effort expectancy. This factor is especially important for new technologies such as mental health chatbot, which is a kind of chatbot that uses AI and natural language to give emotional help and advice to users who struggle with mental health problems (10). Mental health chatbot has advantages such as being available, anonymous, cheap, and customised, but they also have difficulties such as technical problems, ethical dilemmas, and user opposition (80). Previous research has shown that effort expectancy is a big influence on users' intention to use and adopt a mental health chatbot. For instance, the ideas of UTAUT and TPB to explore users' intention to use a mental health chatbot among LGBTQIA+ people (81). They discovered that effort expectancy had a positive impact on attitude, which then affected behavioural intention. They proposed that users who think a mental health chatbot is easy to use are more likely to have a positive attitude towards it and plan to use it in the future. Likewise, the UTAUT model was used to analyse the acceptance and use of chatbots among students in the UK universities (80). They revealed that effort expectancy had a direct positive impact on behavioural intention, as well as an indirect impact through performance expectancy. They claimed that users who think chatbots are easy to use are more likely to anticipate better performance from them and want to use them again.

### Social influence

The opinions of others can shape the users' choices and actions towards using technology, especially when they are not sure about their own likes or skills. The factors that affect the opinions of others include whether the use of technology is optional, how experienced the users are, and what their gender is (79). The opinions of others also matter for a mental health chatbot, which is a kind of chatbot that uses AI and natural language to offer emotional help and advice to users who struggle with mental health problems (10). Some studies have examined the impact of the opinions of others on users' intention to use and adopt a mental health chatbot. For instance, the current state of digital mental health solutions with an emphasis on AI-powered chatbots (82). They claimed that the opinions of others can be a positive factor for users to use a mental health chatbot, as they can get suggestions and feedback from others who have benefited from them. They also mentioned that the opinions of others can be a negative factor for users to shun mental health chatbots, as they can face shame and prejudice from others who do not agree with their use of technology for mental health reasons. Similarly, robots and chatbots can help reduce the mental health crisis caused by the COVID-19 pandemic (83). They discovered that the opinions of others had a positive impact on attitude, which then affected behavioural intention. They proposed that users who think that important others endorse their use of a mental health chatbot are more likely to have a positive attitude towards it and intend to use it in the future.

### Facilitating conditions

Facilitating conditions are about how much a person thinks that they have the needed technical resources and help to use a certain technology. For mental health chatbots, facilitating conditions can involve things such as internet access, device compatibility, chatbot availability, user guidance, and technical assistance. Facilitating conditions can affect the behavioural intention to use mental health chatbots by changing the perceived ease of use and confidence of users (60). Some studies have looked into the role of facilitating conditions in the use of mental health chatbots. For example, facilitating conditions had a positive and important effect on the behavioural intention and usage behaviour of online health community users towards chatbots (84). They recommended that giving clear instructions, feedback, and troubleshooting mechanisms could improve the facilitating conditions for chatbot use. Likewise, facilitating conditions had a direct effect on the behavioural intention to use a non-directive reflective coaching chatbot among professional employees (85). They claimed that making sure reliable internet connection, device compatibility, chatbot availability, and user support could raise the chance of chatbot use. However, facilitating conditions may not always have a strong or direct effect on the behavioural intention to use mental health chatbots. For instance, facilitating conditions had no important effect on the behavioural intention to use Happify Health's AI chatbot, Anna, among adults with depression (82). They said that this could be because of the high availability and accessibility of chatbots through various platforms and devices, which lowered the importance of facilitating conditions as a factor of chatbot use. Moreover, they mentioned that other factors, such as performance expectancy and perceived benefits, could have a stronger effect on the behavioural intention to use mental health chatbots.

### Attitude

Attitude, a basic concept in both UTAUT and TPB, has a crucial role in forming individuals' behavioural intentions towards using technology. Attitude is affected by two main factors: perceived usefulness and perceived ease of use (86). Perceived usefulness is about individuals' beliefs on how using a certain technology can improve their performance and productivity (79, 86). This belief is based on the expectation that the technology will offer real benefits and add value to their work or daily life. On the other hand, perceived ease of use is about individuals' beliefs on how much effort is needed to use a specific technology. When a technology is seen as easy to use, individuals are more likely to have a positive attitude towards it. The opinions of important others, such as family, friends, and colleagues, can influence individuals' attitudes towards using technology. Personal innovativeness measures individual differences in their readiness to adopt new technologies and engage in creative behaviours (87). Individuals with a higher level of personal innovativeness are more likely to show positive attitudes towards using technology. In the UTAUT–TPB integration framework, these different aspects of attitude are studied in relation to other variables to reveal their combined effect on technology acceptance and usage. By treating attitude as a complex concept and examining its relationships with perceived usefulness, ease of use, compatibility, subjective norms, and personal innovativeness, researchers can gain a more complete understanding of the role attitude has in forming individuals' behavioural intentions towards using technology.

### Perceived benefits

Perceived benefits are the positive results or gains that a person hopes to achieve from using a certain technology. For mental health chatbots, perceived benefits can involve perceived usefulness, perceived enjoyment, and perceived trust. Perceived usefulness is about how much a person thinks that using a chatbot can improve their mental health or well-being. Perceived enjoyment is about how much fun or satisfaction a person gets from interacting with a chatbot. Perceived trust is about how much a person trusts that a chatbot is dependable, safe, and ethical (15). Based on the literature, perceived benefits can affect users' intention to use mental health chatbots in different ways. For example, the current state of mental health chatbots found that users appreciated chatbots' personality, character design, conversation style, and content (82). They also found that users’ “perceptions of chatbots” benefits and readiness to use them were affected by their confidence in them. The ideas of UTAUT and TPB to explore users' VR tourism behaviour intention (88). They added perceived benefits as a link between UTAUT and behavioural intention, and attitude as a factor that changes the relationships between UTAUT, perceived benefits, and behavioural intention. They found that UTAUT influenced behaviour through perceived benefits, and attitude positively enhanced these relationships. Therefore, perceived benefits are a key factor to think about when designing and evaluating mental health chatbots for different groups and contexts. By knowing users' needs and preferences, chatbot developers can make chatbots that can offer more useful, enjoyable, and trustworthy services for mental healthcare.

### Behavioural intention

Behavioural intention, a crucial factor in the adoption of mental health chatbots, is influenced by several key considerations. Recent research has provided deeper insights into these factors, shedding light on the users' readiness to engage with such technologies and the specific aspects that shape their intentions. Users need to believe that the chatbot possesses the necessary knowledge and can provide empathetic support. A randomised controlled trial that demonstrated the significance of trust in the chatbot's capabilities (89). Users who trust the chatbot to understand their concerns and offer relevant assistance are more inclined to express a positive behavioural intention to use the technology. The importance of users perceiving the chatbot as a source of emotional support (90). When users believe that the chatbot can offer understanding, empathy, and a sense of connection, their behavioural intention to engage with the technology is likely to increase. This highlights the role of perceived social support in shaping users' intentions. Additionally, users' perception of empowerment and autonomy in their interactions with the chatbot can significantly impact their behavioural intention. Users who perceive the chatbot as empowering and enabling active participation in their mental well-being are more motivated to engage with the technology (89). When users feel they have control over their interactions and can make informed decisions, their intention to use the chatbot increases. Understanding these factors related to behavioural intention in the context of mental health chatbots provides valuable insights for the design, implementation, and promotion of effective interventions. By considering factors such as confidentiality and privacy, trust in chatbot capabilities, perceived social support, and user empowerment, developers can create chatbots that align with users' needs, fostering positive behavioural intentions and promoting mental well-being.

### Theoretical framework

The research project merges the theories of the Theory of Planned Behaviour (TPB) and the Unified Theory of Acceptance and Use of Technology (UTAUT) to investigate the variables that affect professional employees' behavioural intention to utilise mental health chatbots. The UTAUT model describes how four variables—performance expectancy, effort expectancy, social influence, and facilitating conditions—affect a person's behaviour with regard to using a technology. According to the TPB model, human behaviour is influenced by three variables: attitude, subjective norm, and perceived behavioural control. Perceived benefits and attitude towards chatbots are additional variables that the study considers. The advantages or favourable results that a person expects to obtain from utilising a certain technology are known as perceived benefits. Attitude towards chatbots is the positive or negative evaluation that a person has towards using a chatbot for mental health.

**Figure 2:**
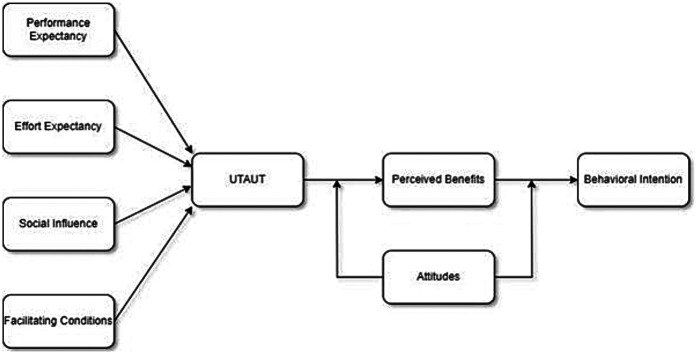
The proposed model of integrated concepts of UTAUT and TPB for mental health chatbot behaviour intention.

According to this research's hypothesis, UTAUT variables positively and significantly affect perceived benefits, which in turn positively and significantly affect behavioural intention to use mental health chatbots. The study also makes the following hypotheses: attitude towards chatbots moderates the correlations between UTAUT components, perceived benefits, and behavioural intention, and perceived benefits mediate the relationship between UTAUT factors and behavioural intention. The study further examines how user characteristics such as gender, age, experience, and usage have a moderating effect on these relationships. The proposed theoretical framework is similar to the one used by Huang (88), who integrated UTAUT and TPB to investigate users' VR tourism behaviour intention. They also added perceived benefits as a mediator and attitude as a moderator between UTAUT factors and behavioural intention. However, this study differs from them in that it focuses on mental health chatbots instead of VR tourism, and it targets professional employees instead of general consumers. See Figure 2 for the proposed integrated model of UTAUT and TPB.

The UTAUT and TPB are combined by using the UTAUT factors as the precursors of perceived benefits, which is a mediator between UTAUT factors and behavioural intention. The TPB factor attitude is used as a moderator between UTAUT factors, perceived benefits, and behavioural intention. This means that the TPB factor attitude can influence how strongly the UTAUT factors affect perceived benefits and how strongly perceived benefits affect behavioural intention. This TPB variable is not shown as a direct predictor of behaviour in the proposed theory. Instead, it is shown as a moderator between the UTAUT variables, perceived benefits, and behavioural intention. This means that it can influence how strongly the UTAUT variables affect perceived benefits and how strongly perceived benefits affect behavioural intention. For example, if a person has a positive attitude towards chatbots, they may perceive more benefits from using a mental health chatbot and have a higher intention to use it. Conversely, if a person has a negative attitude towards chatbots, they may perceive fewer benefits from using a mental health chatbot and have a lower intention to use it.

### Hypothesis development

According to the previous research analysis, UTAUT elements may affect users' acceptance and adoption of chatbots and other technologies. The advantages or favourable outcomes that consumers anticipate getting from using a certain technology are known as perceived benefits. The perception that people feel about utilising a chatbot for mental health might be favourable or adverse, depending on their attitude towards chatbots. The extent to which a user plans or intends to utilise a certain technology in the future is known as behavioural intention. UTAUT variables, perceived benefits, attitude towards chatbots, and behavioural intention are connected and can influence one another in various ways, according to a number of previous studies (22). In the context of mental health chatbots for professional employees, this study hypothesises that these factors have direct, indirect, and moderating impacts on each other. Both a survey approach and a quantitative research design will be used to evaluate these assumptions. The results will have impacts for theory and practice and give insights into the acceptance and implementation of mental health chatbots among professional employees.

### The relationship of UTAUT factors on perceived benefits

The theoretical framework proposes a correlation between UTAUT factors and perceived benefits. The UTAUT model states that four primary factors significantly impact an individual's behavioural intention to adopt and use a technology. These factors include performance expectancy, effort expectancy, social influence, and facilitating conditions. Perceived benefits refer to the anticipated favourable consequences or advantages that an individual expects to gain from the utilisation of a particular technology. The UTAUT model identifies factors that have a direct or indirect impact on behavioural intention, mediated by perceived benefits (74). Consequently, a hypothesis has been formulated that the UTAUT factors exert a beneficial and significant impact on the perceived benefits of mental health chatbot usage among individuals employed in professional settings. The previous statement suggests that the level of perceived benefits of utilising mental health chatbots is directly related to the degree of performance expectancy, effort expectancy, social influence, and facilitating conditions associated with their usage. This relationship is supported by previous studies that have found that UTAUT factors are positively associated with perceived benefits in various contexts and technologies (75, 78), as below:

*H1: UTAUT factors have a positive and significant influence on perceived benefits.*

Professional employees may anticipate multiple benefits from utilising mental health chatbots, such as better mental health outcomes, enhanced access to information and support, decreased stigma and cost, and improved self-management and empowerment. The advantages of utilising mental health chatbots, such as convenience, affordability, and confidentiality, may serve as incentives for professional employees to seek assistance for their mental health concerns. Furthermore, it is significant that each factor of UTAUT may exert varying effects on the perception of benefits. The perceived benefits of mental health chatbots may be influenced by various factors. Particularly, performance expectancy may impact the perceived usefulness and effectiveness of these chatbots, while effort expectancy may affect their perceived ease and convenience. Additionally, social influence may play a role in shaping perceptions of the acceptability and desirability of mental health chatbots, and facilitating conditions may influence their perceived availability and compatibility. The previously mentioned variables have the potential to influence the perceptions of mental health chatbots' value and suitability among professional employees, based on their individual needs and preferences (91).

### The relationship of perceived benefits on behavioural intention

The relationship between perceived benefits and behavioural intention is an important aspect of the theoretical framework, as it relates to the impact of perceived benefits on behavioural intention. Perceived benefits refer to the anticipated favourable consequences or advantages that an individual intends to receive from the utilisation of a particular technology. Behavioural intention refers to an individual's intention or desire to utilise a specific technology in the future. As per the UTAUT model proposed by Venkatesh and Davis (86), the impact of perceived benefits on behavioural intention can be observed either directly or indirectly through other influencing factors (86). Hence, a hypothesis has been put forth that the perceived benefits of mental health chatbot usage have a constructive and significant impact on the behavioural intention of professional employees. The previously mentioned hypothesis suggests that an increase in the perceived advantages associated with the utilisation of mental health chatbots will correspondingly result in an elevated tendency to engage with them. This hypothesis is strengthened by prior research, which has established a positive correlation between perceived benefits and behavioural intention across diverse technologies and contexts (81, 92), as below:

*H2: Perceived benefits have a positive and significant influence on behavioural intention.*

The impact of perceived benefits on behavioural intention can be attributed to the perceived value and worth of mental health chatbots among professional employees. If mental health chatbots are perceived by professional employees to be an opportunity to enhance their mental health outcomes, increase their access to information and support, reduce stigma and cost, and improve their self-management and empowerment, they will likely be more inclined to utilise them in the future (91). The influence of perceived benefits on behavioural intention can be attributed to the compatibility and suitability of mental health chatbots as perceived by professional employees in relation to their needs and preferences. If mental health chatbots are perceived by professional employees to align with their lifestyle, work environment, personal goals, and values, they will likely exhibit a greater willingness to utilise them in the future.

### The mediating relationship of perceived benefits on the relationship between UTAUT and behavioural intention

The theoretical framework incorporates the concept that perceived benefits serve as a mediator in the relationship between UTAUT factors and behavioural intention. According to the proposal of Baron and Kenny (112), a mediating effect occurs when an independent variable affects a dependent variable through another variable, clarifying the mechanism or rationale behind the effect. This study examines the impact of UTAUT factors as independent variables on the dependent variable of behavioural intention. The mediating variable that explains the impact of UTAUT factors on behavioural intention is perceived benefits. Perceived benefits refer to the favourable consequences or advantages that an individual expects to derive from the utilisation of a technological tool. According to Venkatesh and Davis (86) (91), the UTAUT model proposes that the impact of UTAUT factors on behavioural intention can be mediated by perceived benefits. The present research suggests that the mediating effect of perceived benefits is positive and significant in the relationship between UTAUT factors and behavioural intention in the context of mental health chatbot usage among professional employees. The previous statement suggests that the perceived benefits serve as a mediator in clarifying the indirect impact of UTAUT factors on the probability of adoption of mental health chatbots by people employed in professional settings. This statement is supported by previous studies that have identified the mediating effect of perceived benefits on the impact of UTAUT factors on behavioural intention across various technologies and contexts.

*H3: Perceived benefits have a positive, mediating effect on the relationship between UTAUT and behavioural intention.*

The relationship between UTAUT elements and behavioural intention may be mediated by perceived advantages, which explains how UTAUT factors influence behavioural intention through perceived benefits. For instance, performance expectancy may affect the perception of the value and benefit of mental health chatbots, which may then influence how likely professional employees are to use them in the future (113). This perception may be influenced by how useful and effective mental health chatbots are perceived to be. In similar ways, effort expectancy may affect behavioural intention by influencing how simple and practical mental health chatbots are perceived to be; this perception may then influence how compatible and suitable mental health chatbots are perceived; and finally, this perception may influence how willing professional employees are to use them in the future (93, 94). The same rationale applies to facilitating conditions and social influence. Perceived advantages may therefore serve as the link connecting UTAUT variables and behavioural intention.

#### The moderating relationship of attitude towards chatbot on the relationships between UTAUT and perceived benefits

According to the moderating effect of chatbot attitude on the relationships between UTAUT and perceived benefits, a person's opinion of using a chatbot for mental health can affect how strongly UTAUT factors affect perceived benefits and how strongly perceived benefits affect intention to use the chatbot. A person may see greater benefits from utilising a chatbot for mental health, for instance, if they have a favourable attitude towards them, they may be more likely to use one. On the other hand, if a person has an unfavourable viewpoint towards chatbots, they might see fewer benefits to using a mental health chatbot and be unwilling to use it. Grové (77) looked at the acceptability and efficiency of a chatbot for mental health that was co-developed with and for young people and found evidence to support this effect. For establishing their research objectives and data analysis, she used the TPB as a theoretical framework. She discovered that the intention of young people to use the chatbot was significantly influenced by attitude. Young people appreciated the chatbot's personality, character design, conversation style, and content. Another study that confirmed this result was carried out by Huang (88), who combined UTAUT and TPB and attitude as a mediator to gain insight into users' intention to engage in VR tourism.

*H4: Attitudes towards chatbots positively strengthen the relationships between UTAUT and perceived benefits.*

The way in which UTAUT factors are viewed and assessed by professionals could influence the relationship between these parameters and perceived advantages. Depending on whether professional employees have a favourable or unfavourable attitude towards chatbots, for instance, performance expectancy may have a distinct impact on perceived advantages. Professional employees may view mental health chatbots as more helpful and effective if they view them positively than if they view them unfavourably, and this perspective may then influence how important and worthwhile mental health chatbots are perceived (114). Similarly, depending on whether professional employees have a favourable or unfavourable attitude towards chatbots, effort expectancy may have a distinct impact on perceived benefits (95). Professional employees may view mental health chatbots as simpler to use and convenient if they have a favourable attitude towards chatbots than if they have a negative attitude; this perspective may then influence how compatible and appropriate mental health chatbots are thought to be. The same reasoning holds true for facilitating conditions and social influence. As a result, one factor that alters the strength or direction of the association between UTAUT parameters and perceived benefits is attitude towards chatbots.

#### The moderating relationship of attitude towards chatbot on the relationships between perceived benefits and behavioural intention

The relationship between perceived benefits and behavioural intention is moderated by chatbot attitude, which is the fifth relationship in the theoretical framework. The way a person feels about using a chatbot for mental health might be either favourable or negative. Perceived benefits are the advantages or favourable results that a person anticipates gaining from employing a specific technology. The level of future technology use that a person intends or desires to make use of is known as behavioural intention. According to the UTAUT model (79, 95), a chatbot's attitude may influence the relationship between perceived benefits and behavioural intention. Therefore, it is hypothesised that among professional employees who utilise mental health chatbots, attitude towards the chatbot has a positive and significant moderating effect on the relationship between perceived benefits and behavioural intention. This means that attitude towards chatbots affects how perceived benefits influence behavioural intention. According to another research (75), attitude towards chatbots moderates the link between perceived advantages and behavioural intention in a variety of situations and technologies.

*H5: Attitudes towards chatbots positively strengthen the relationships between perceived benefits and behavioural intention.*

Through having an effect on how perceived benefits are interpreted and evaluated by professionals, attitude towards chatbots may alter the relationship between behavioural intention and perceived benefits (96). If professional employees have a favourable or unfavourable attitude towards chatbots, for instance, perceived benefits may influence behaviour intention differently. If professional employees have a positive attitude towards chatbots, they may perceive mental health chatbots as more valuable and worthwhile than if they have a negative attitude towards chatbots; this perception may then affect how likely and willing professional employees are to use them in the future (97). Likewise, depending on whether professional employees have a favourable or unfavourable view towards chatbots, perceived benefits may have a unique effect on behavioural intention. If professional employees have a positive attitude towards chatbots, they may perceive mental health chatbots as more compatible and suitable than if they have a negative attitude towards chatbots; this perception may then affect how willing and able professional employees are to use them in the future. As a result, one factor that alters the strength or direction of the link between perceived advantages and behaviour intention is attitude towards chatbots.

### Overview of past and current literature

An overview of the previous and present literature on users' desire to utilise chatbots for various situations and purposes is given in this section. It summarises the objectives, methods, theories, and findings of research projects that have been carried out in various nations and sectors. Additionally, it highlights frequent themes, gaps, and constraints in the available literature and provides suggestions for future study topics. The Unified Theory of Acceptance and Use of Technology (UTAUT) or its extension (UTAUT2) served as the common theoretical framework in a significant amount of research used to explain and forecast users' intention to use chatbots. Some research has included the UTAUT model with elements of the Theory of Planned Behaviour (TPB), such as attitude, subjective norm, and perceived behavioural control. Other factors that were included in some research were perceived benefits, attitudes towards chatbots, cognitive absorption, risk, habit, and COVID-19 impact (98). Data collection and analysis methods employed in the investigations included surveys, interviews, experiments, and structural equation modelling.

The outcomes of the studies demonstrated that a mix of personal, psychological, and technical elements affected people's intentions to utilise chatbots. Performance expectation, effort expectancy, social influence, perceived benefits, and attitude were the most frequent variables that had a positive and substantial impact on intention. The correlations between other variables and intention were also influenced by some factors in a moderating or mediating manner. For instance, user characteristics such as gender, age, experience, and usage moderated the effects of various factors on intention, and attitude towards chatbots moderated the effects of UTAUT factors on perceived benefits and intention. Perceived benefits also mediated the effects of UTAUT factors on intention. The research also identified several gaps and constraints in the body of knowledge on users' motives for using chatbots. For example, most of the research focussed on chatbots in e-commerce or healthcare contexts, whereas other areas such as education or entertainment received less attention. A qualitative method might offer more information on users' perspectives and experiences with chatbots than the majority of research, which employed a quantitative technique to assess users' intention to use chatbots. Furthermore, most studies did not evaluate the actual use behaviour or the outcomes of using chatbots for users' mental health or well-being. Therefore, future research should explore different contexts and objectives for using chatbots, employ several methods to gather and evaluate data, and analyse actual use behaviour and results to overcome these gaps and constraints. See Table 1 for a summary of related studies and findings.

**Table 1:** Summary of related studies.

Reference	Author (year)	Objective	Findings
(96)	Gatzioufa and Saprikis (2022)	Examine chatbot acceptability and effectiveness for young people	Attitude, subjective norm, and perceived behavioural control significantly predicted intention to use chatbot
(81)	Henkel et al. (2022)	Examine chatbot preferences for LGBTQIA+ users	Performance expectancy, effort expectancy, social influence, perceived benefits, and attitude significantly predicted intention to use chatbot. Perceived benefits mediated UTAUT and TPB variables and intention. Attitude moderated UTAUT variables, perceived benefits, and intention
(99)	Francis (2019)	Examine data acceptance from patient self-monitoring devices by healthcare professionals	Performance expectancy, effort expectancy, social influence, and enabling settings significantly predicted intention to use data. Use behaviour influenced by intention and habit. Age and gender mediated some relationships
(79)	Venkatesh et al. (2003)	Incorporate consumer perspective into UTAUT	Hedonic motivation, price value, and habit added to UTAUT. Model explained 52% of use and 74% of intention. Age, gender, and experience moderated some effects
(78)	Ball et al. (2021)	Examine factors affecting virtual reality adoption for mental health	Performance expectancy, effort expectancy, social influence, perceived behavioural control, perceived benefits, and attitude significantly predicted intention to use VR. Perceived benefits mediated UTAUT variables and intention. Attitude moderated UTAUT variables, perceived benefits, and intention
(26)	Abd-Alrazaq et al. (2021)	Examine usability and functionality satisfaction of chatbot Anna by university students	Users rated Anna high on usability and satisfaction. Anna provided relevant advice and support for various mental health conditions
(100)	Balakrishnan and Dwivedi (2021)	To examine human- to-machine and human-to-human interaction effects on service outcomes	Human-to-machine interaction enhanced cognitive absorption, trust, experience, and continuation intention more than human-to-human interaction
(101)	Adamopoulou and Moussiades (2020)	Investigate factors affecting chatbot adoption for e-commerce	Perceived usefulness, perceived ease of use, attitude, subjective norm, and perceived behavioural control significantly predicted intention to use chatbot. Perceived risk and trust moderated these relationships
(102)	Gupta et al. (2018)	Discover variables affecting smartphone travel app adoption by visitors	Simplicity of use, assistance, and enjoyment did not influence users’ tendency to use travel apps while benefit, social influence, cost savings, risk, and habit did
(78)	Ball et al. (2021)	Find out how VR devices were used and adopted during the epidemic	COVID-19 impact affected the probability of purchasing VR for business, travel, and education. Social interactivity anticipated VR gadget use and buying desires. Leisure and games were the most widely used VR applications during the epidemic, by using the uses and gratifications theory (UGT).
(80)	Almahri et al. (2020)	Analyse student acceptance and use of chatbots at institutions using SEM with the UTAUT2 model	Behavioural intention to use chatbots influenced by performance expectation, effort expectancy, social influence, enabling situations, hedonic motivation, and habit. Use behaviour influenced by intention and habit. Experience and gender mediated some correlations
(103)	Chocarro et al. (2021)	Examine instructors’ intention to adopt virtual agents in education using TAM perspective	Chatbots’ perceived utility and usability had a favourable impact on usage intentions. Use of social language and emoticons negatively impacted the desire to utilise. Instructors’ age, digital literacy, and chatbot proactivity did not affect intention to use
(92)	Lipschitz et al. (2019)	Investigate patient interest in and engagement concerns with mobile apps for anxiety and depression	Patients expressed strong desire to use mobile apps to treat their depression and anxiety but identified barriers such as expense, lack of support, privacy concerns, and low self-efficacy
(104)	Tarhini et al. (2016)	Examine elements that may hinder or facilitate online banking adoption	Expectancy, social influence, perceived credibility, and task–technology fit had substantial impact on behavioural intention, but effort expectation did not. Facilitating conditions and behavioural intention significantly influenced actual usage

## Research methodology

### Research design

This study adopts a quantitative approach, utilising structured questionnaires to collect data at a singular point in time through a cross-sectional survey methodology. This method is instrumental in performing statistical analyses, testing hypotheses, and exploring correlations among the study variables.

### Target population and sampling strategy

The target population for this investigation comprises individuals in Malaysia diagnosed with mental health issues, as they represent the potential primary users of mental health chatbots. An initial screening mechanism was built into the online survey to ensure that the sample met the study's objectives. Respondents had to meet the following inclusion criteria: (1) currently working as a professional in Malaysia and (2) having prior experience with or interest in mental health chatbots or digital health apps. Incomplete responses, inability to pass attention-check items, and failing to meet the inclusion criterion were all grounds for exclusion. Only individuals who met the screening criteria were able to advance to the main questioning. To ensure that participants met the inclusion criteria, two screening questions were incorporated at the beginning of the survey: (1) “Are you currently working as a professional in Malaysia?”, and (2) “Do you have prior experience with or interest in mental health chatbots or digital health applications?” These questions were designed to confirm both professional status and relevance to the study topic. All respondents who proceeded to the main questionnaire satisfied these criteria, and no participants were excluded or dropped out due to the exclusion conditions.

Given the study's targeted demographic, a refined screening process is implemented to identify and select participants accurately. This process involves using convenience sampling, a non-probability sampling technique that relies on the accessibility of participants. While this method enables swift and cost-effective data collection from a wide array of respondents fitting the study's criteria, it also necessitates a meticulous filtering mechanism to ensure participants align with the research objectives. To accurately target respondents diagnosed with mental health issues, the initial section of the online survey includes screening questions designed to ascertain the participants' mental health status. These questions are crafted based on recognised diagnostic criteria and are intended to confidentially verify whether respondents have been diagnosed with or are experiencing symptoms of mental health conditions.

### Sample size determination

The G*Power analysis indicated a minimum sample size of 98 participants to achieve adequate statistical power. However, data collection continued until 351 valid responses were obtained. This number was not arbitrary; it was determined to enhance the robustness of the structural equation modelling (SEM) analysis, allow for subgroup comparisons, and mitigate potential issues related to non-response or incomplete data. Additionally, practical considerations such as time constraints and resource availability influenced the decision to conclude data collection at this point. The final sample size exceeds the minimum requirement, thereby improving the reliability and generalisability of the findings.

### Data collection procedure

Data analysis employs Structural Equation Modelling (SEM) via SmartPLS software, adept at managing intricate models comprising both latent and observed variables. This analytical method is particularly suitable for exploratory research involving smaller sample sizes, allowing for an in-depth examination of the factors influencing mental health chatbot usage intentions among professionals diagnosed with mental health issues. This tailored approach ensures the research directly addresses the interaction between mental health challenges and the propensity to engage with chatbot technology for support. Prior to participation, all respondents were provided with detailed information about the study and gave their informed consent voluntarily, ensuring ethical compliance and respect for participant autonomy.

### Measures

The survey instrument for this study is a questionnaire that consists of two sections as shown in Appendix A. The first section collects demographic information of the respondents, such as gender, age, education level, occupation, and experience with chatbots. The second section uses a 5-point Likert scale to measure the variables of the study, namely, UTAUT factors (performance expectancy, effort expectancy, social influence, and facilitating conditions), perceived benefits, attitude towards chatbot, and behavioural intention to use a mental health chatbot. The questionnaire items were adapted from previous studies that used UTAUT and TPB models to examine users' intention to use chatbots or other technologies.

### Survey instrument

The items included in the questionnaire for this study were derived from established and validated measurement scales to ensure both reliability and alignment with prior research. Constructs from the Unified Theory of Acceptance and Use of Technology (UTAUT)—namely, performance expectancy, effort expectancy, social influence, and facilitating conditions—were adapted from the widely cited work of Venkatesh et al. (79), commonly used in studies on technology adoption. Items assessing perceived benefits were based on the technology acceptance model (105) and refined using insights from health-related technology adoption studies (105, 106). Attitudes towards chatbots were measured using elements from the Theory of Planned Behaviour (76), supplemented with items from recent studies on chatbot usage [e.g., (107)]. Behavioural intention was evaluated using items adapted from Venkatesh et al. (79) and other relevant literature on technology acceptance, aiming to capture participants' willingness to use mental health chatbots.

By employing these well-established scales, each demonstrating strong reliability in previous studies, the study ensures the robustness of its measurements and enhances the comparability of its findings with existing research in the field.

## Research findings

### Demographic profile of the respondents

The finding shows that a majority of respondents (76.7%) are primarily likely to utilise mental health chatbots for information or advice on mental health issues, coupled with the substantial likelihood (75%) of future usage. This reflects in the demographic profile of the respondents, suggesting that users are satisfied with their experience or perceive the chatbot as a useful and convenient tool for their mental health needs. In this study, the demographic analysis reveals a nearly equal gender distribution among the respondents, with 49.2% male and 50.8% female, suggesting that both men and women are equally interested and willing to use a mental health chatbot. Most participants fall within the 25–34 age group (38.3%), followed by the 35–44 age bracket (27.5%), which indicates that young professionals are more likely to use a mental health chatbot, perhaps due to their familiarity with technology, their busy lifestyles, or their higher exposure to stress and mental health challenges. Regarding education, half hold a bachelor's degree, while 25.8% have a master's degree. This shows that mental health chatbots appeal to well-educated users, who may have higher expectations and standards for the quality and reliability of the chatbot. The majority are engineers (34.2%), followed by teachers (25.8%) and accountants (14.2%). The diverse professional fields of the respondents, with engineers, teachers, and accountants being the most common, demonstrate that mental health chatbots can cater to different occupational backgrounds and needs, as well as different levels of income and social status. Most have 1–3 years of work experience (38.3%), which implies that mental health chatbots may be more suitable for new or young workers, who may face more challenges and uncertainties in their careers, or who may have less access to other sources of mental health support. See Table 2 for a detailed summary of the demographic profile of the respondents.

**Table 2:** Summary of demographic profile of the respondents.

Demographic trait	Characteristics	Frequency	Percentage
Gender	Male	160	45.58
	Female	191	54.42
Age group	Below 24	20	5.7
	25–34	106	30.2
	35–44	99	28.2
	45–54	85	24.2
	55 and above	41	11.7
Highest education level	High school diploma	89	25.4
	Bachelor's degree	120	34.2
	Master's degree	99	28.2
	Doctoral degree	43	12.2
Current occupation	Accountant	60	17.1
	Doctor	58	16.5
	Engineer	81	23.1
	Manager	55	15.7
	Teacher	70	19.9
	Other	27	7.7
Working experience	<1 year	32	9.1
	1–3 years	106	30.2
	4–6 years	86	24.5
	7–9 years	61	17.4
	10 years and above	66	18.8
Main reason for using a chatbot for mental health	To cope with stress, anxiety, depression, or other mental health issues	87	24.8
	To get emotional support or comfort	101	28.8
	To get information or advice on mental health issues	163	46.4
How likely are you to use a chatbot for mental health in the future	Very Likely	99	28.2
	Likely	82	23.3
	Neutral	77	21.9
	Unlikely	50	14.3
	Very Unlikely	43	12.3

### Measurement model results

**Figure 3:**
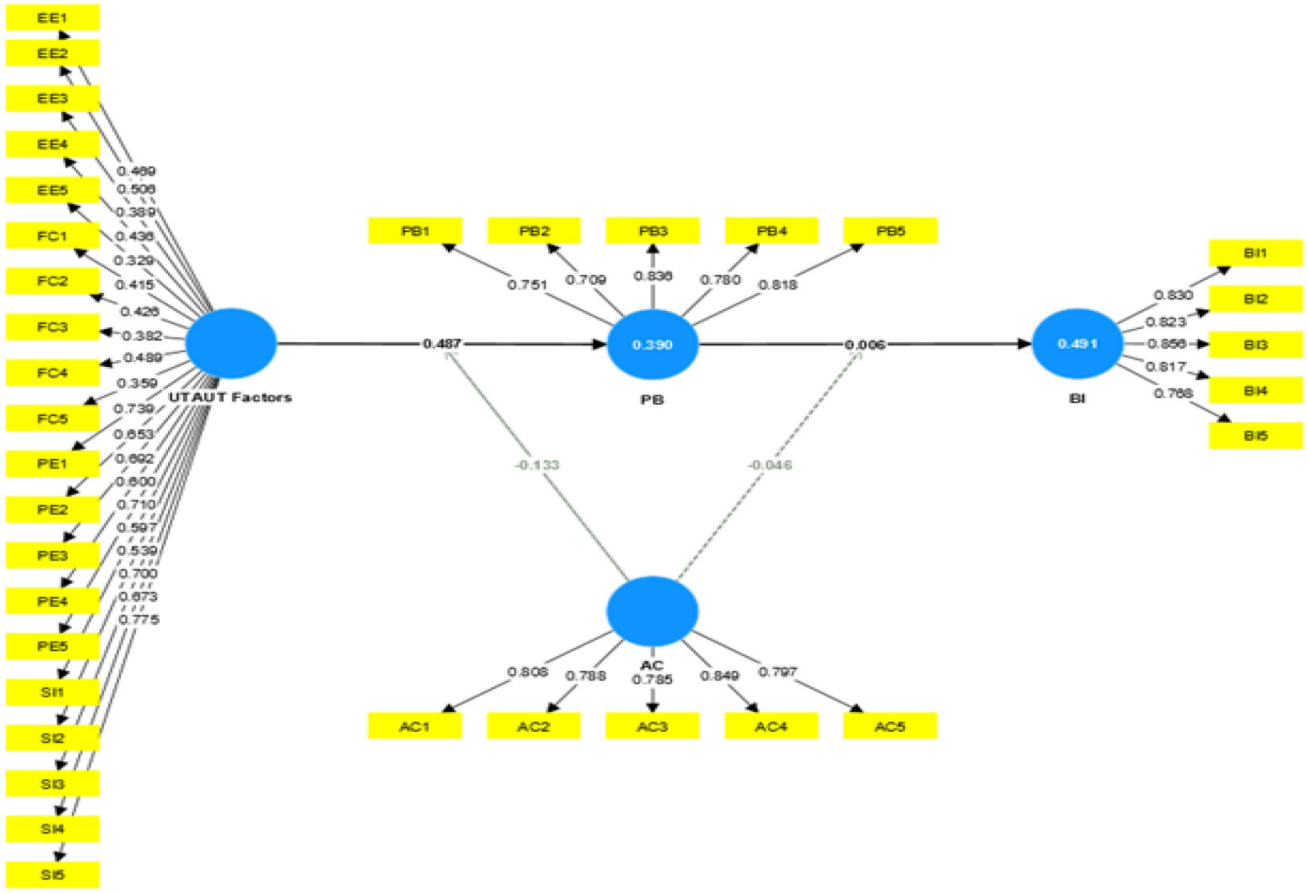
Structural model.

In this study's measurement model, the latent variables were assessed using various items, each with its loading value. For UTAUT factors, items ranged from PE1 to FC5, showing varying degrees of loading, with the highest being SI5 at 0.775. The composite reliability (CR) and Cronbach's alpha (CA) for these factors indicate good internal consistency, although the average variance extracted (AVE) values are relatively low. In the perceived benefits category, items PB1 to PB5 showed strong loadings, with PB3 being the highest at 0.836, reflecting a robust construct. The attitude construct, with items AC1 to AC5, also displayed high loadings, notably AC4 at 0.849. Finally, the behavioural intention category, with items BI1 to BI5, demonstrated strong loadings, particularly BI3 at 0.856, signifying a solid connection to the latent variable. The overall measurement model results reflect a reliable and valid construct for analysing the factors influencing behavioural intention to use mental health chatbots. See Table 3 for detailed measurement model results. Figure 3 shows the outcomes of the structural model.

**Table 3:** Measurement model results.

Latent variable	Item	Mean	SD	Loading	CA	CR	AVE
UTAUT factors	PE1	3.908	0.992	0.739	0.899	0.896	0.315
	PE2	3.808	0.906	0.653			
	PE3	3.867	0.912	0.692			
	PE4	3.992	0.890	0.600			
	PE5	3.892	0.998	0.710			
	EE1	3.858	0.849	0.469			
	EE2	3.900	1.020	0.506			
	EE3	3.767	1.014	0.389			
	EE4	3.833	0.943	0.436			
	EE5	3.683	0.949	0.329			
	SI1	3.575	1.070	0.597			
	SI2	3.692	1.094	0.539			
	SI3	3.583	1.242	0.700			
	SI4	3.758	0.931	0.673			
	SI5	3.650	1.100	0.775			
	FC1	3.692	1.131	0.415			
	FC2	3.658	1.037	0.426			
	FC3	3.767	1.047	0.382			
	FC4	3.800	1.005	0.489			
	FC5	3.800	1.030	0.359			
Perceived benefits	PB1	3.925	0.941	0.751	0.839	0.886	0.608
	PB2	3.775	0.970	0.709			
	PB3	3.867	0.957	0.836			
	PB4	3.833	0.869	0.780			
	PB5	3.850	0.989	0,818			
Attitude	AC1	3.933	0.873	0.808	0.865	0.902	0.649
	AC2	3.767	0.929	0.788			
	AC3	3.625	1.009	0.785			
	AC4	3.917	0.997	0.849			
	AC5	3.86	1.008	0.797			
Behavioural intention	BI1	3.733	0.896	0.830			
	BI2	3.825	0.980	0.823			
	BI3	3.725	1.140	0.856			
	BI4	3.767	0.964	0.817			
	BI5	3.675	0.808	0.768			

SD, standard deviation; CR, composite reliability; CA, Cronbach's alpha; AVE, average variance extracted.

### Discriminant validity of constructs

The Fornell–Larcker results in this research indicate the discriminant validity of the constructs, attitude towards use (AC), behavioural intention (BI), perceived benefits (PB), and UTAUT factors as shown in Table 4. Each construct demonstrates a strong association with itself, evidenced by the diagonal values (AC, 0.806; BI, 0.819; PB, 0.780; UTAUT factors, 0.561). The off-diagonal values, representing inter-construct correlations, are lower than the diagonal values, supporting the distinctiveness of the constructs. Particularly, AC and BI show a moderate correlation, while the link between PB and other factors is comparatively weaker.

**Table 4:** Fornell–Larcker results.

Constructs	AC	BI	PB	UTAUT factors
AC	0.806			
BI	0.697	0.819		
PB	0.363	0.266	0.780	
UTAUT factors	0.596	0.449	0.601	0.561

AC, attitude towards use; BI, behavioural intention; PB, perceived benefits; UTAUT, Unified Theory of Acceptance and Use of Technology.

The heterotrait–monotrait ratio (HTMT) in Table 5 provides insights into the discriminant validity of the constructs. Notably, the strongest relationship is between “AC × UTAUT Factors” and “AC × PB” (HTMT = 0.817), indicating a high degree of correlation. The “AC” and “BI” constructs also show a substantial correlation (HTMT = 0.781). The relationships between other constructs, such as “PB” and “UTAUT factors” or “AC” and “UTAUT factors”, are moderate, with HTMT values of 0.538 and 0.574, respectively. These results suggest a clear distinction between constructs while also revealing significant interrelationships, crucial for validating the research model.

**Table 5:** Heterotrait–monotrait ratio (HTMT).

Constructs	AC	BI	PB	UTAUT factors	AC–UTAUT factors	AC–PB
AC						
BI	0.781					
PB	0.415	0.308				
UTAUT factors	0.574	0.419	0.538			
AC–UTAUT factors	0.532	0.483	0.536	0.599		
AC–PB	0.442	0.371	0.263	0.526	0.817	

#### Path coefficient and hypothesis summary

In this research, path coefficients are employed to evaluate the significance and relevance of relationships within the structural model. This assessment is pivotal in determining the empirical substantiation of the conceptual model or theoretical hypotheses. The analysis of hypotheses revealed varied outcomes: Hypothesis 1 (H1), positing that UTAUT factors significantly influence perceived benefits, was strongly supported (path coefficient = 0.802, *p* = 0.001). However, the subsequent hypotheses were not supported. Hypothesis 2 (H2), linking perceived benefits to behavioural intention, and Hypothesis 3 (H3), regarding the mediating effect of perceived benefits, showed insignificant influences with respective *p*-values of 0.529 and 0.263. Similarly, Hypotheses 4 (H4) and 5 (H5), examining the role of attitudes towards chatbots in strengthening relationships between UTAUT factors, perceived benefits, and behavioural intention, were not supported, indicated by their respective *p*-values of 0.094 and 0.695. Table 6 shows the summary results of hypothesis statements, and Table 7 shows the summary of re-analysed results.

**Table 6:** Summary of hypothesis statements results.

Hypotheses	Relationships	Path coefficients	*T*-values	*p*-values	Decision
H1	UTAUT factors have a positive and significant influence on perceived benefits.	0.802	3.274	0.001**	Supported
H2	Perceived benefits have a positive and significant influence on behavioural intention.	0.082	0.629	0.529	Not supported
H3	Perceived benefits have a positive, mediating effect on the relationship between UTAUT and behavioural intention.	0.063	1.121	0.263	Not supported
H4	Attitudes towards chatbots positively strengthen the relationships between UTAUT and perceived benefits.	0.063	1.674	0.094	Not supported
H5	Attitudes towards chatbots positively strengthen the relationships between perceived benefits and behavioural intention.	0.183	0.391	0.695	Not supported

**p < 0.001.

**Table 7:** Summary of re-analysed results (removed factor loadings below 0.5).

Hypotheses	Relationships	Path coefficients	*T*-values	*p*-values	Decision
H1	UTAUT factors have a positive and significant influence on perceived benefits.	0.793	4.432	4.26E−13	Supported
H2	Perceived benefits have a positive and significant influence on behavioural intention.	0.107	0.090	0.464	Not supported
H3	Perceived benefits have a positive, mediating effect on the relationship between UTAUT and behavioural intention.	Not available	Not available	Not available	Not available
H4	Attitudes towards chatbots positively strengthen the relationships between UTAUT and perceived benefits.	0.009	1.650	0.094	Not supported
H5	Attitudes towards chatbots positively strengthen the relationships between perceived benefits and behavioural intention.	0.050	0.831	0.203	Not supported

## Discussion

The discussion section of this study interprets the findings in relation to the existing literature, offering insights into the dynamics of professional employees' behavioural intentions towards mental health chatbots. The results are analysed based on the hypotheses tested, and the findings are justified whether supported or not.

*Research Question 1: What is the relationship between UTAUT factors (represented by performance expectancy, effort expectancy, social influence, and facilitating conditions) and perceived benefits among professional employees?*

Hypothesis 1 (H1) that perceived UTAUT factors have a positive and significant influence on perceived benefits is supported (path coefficient = 0.802, *p* = 0.001). The strong positive correlation between UTAUT factors and perceived benefits suggests that the respondents who find a technology to be useful, easy to use, socially endorsed, and supported by adequate resources are more likely to recognise its advantages. This relationship is further reinforced by the significant path coefficient and *p*-value, which indicate a robust statistical link. This finding aligns with existing literature emphasising the impact of UTAUT factors on users' perceptions of technology benefits. For instance, Grové and Henkel et al. (77, 81) found similar positive relationships in different contexts, indicating that when users perceive high performance expectancy, effort expectancy, social influence, and facilitating conditions, they are likely to see more benefits in using a technology, such as a mental health chatbot. This is consistent with the general understanding that when technology meets user expectations in terms of utility and ease of use, and when it is supported socially and by necessary infrastructure, users perceive higher benefits. The demographic analysis suggests that younger professionals (50.0%) are more inclined towards technology adoption, which may enhance the mediation effect of perceived benefits. This implies that for these individuals, the perceived advantages of chatbots—such as accessibility and personalised support—could strongly influence their intention to use such technologies in managing mental health. Consequently, this enhances the likelihood of technology adoption and sustained use within professional settings.

*Research Question 2: What is the relationship between perceived benefits and behavioural intention to use a mental health chatbot among professional employees?*

The study expected that perceived benefits would have a positive and significant influence on behavioural intention, meaning that professional employees who see more benefits in using mental health chatbots would be more willing to use them. However, Hypothesis 2 (H2) that perceived benefits have a positive and significant influence on behavioural intention is not supported (path coefficient = 0.082, *p* = 0.529). Contrary to expectations and existing studies such as those by Lipschitz et al. (92), this study did not find a significant relationship between perceived benefits and behavioural intention. A possible explanation for this discrepancy is that respondents might have different priorities or preferences when it comes to using mental health chatbots, and they might value other factors. For instance, respondents might prioritise factors other than perceived benefits, such as confidentiality or accuracy, more heavily when deciding to use mental health chatbots. The demographic insights suggest that younger professionals (50.0%), who are typically more tech-savvy, may recognise the advantages of chatbots more readily, thus influencing their intention to use such technologies. Conversely, seasoned professionals (22.5%) might weigh the perceived benefits against their established coping mechanisms and preferences for traditional support systems. This implies that technology adoption is a complex and multifaceted phenomenon and that perceived benefits alone might not be enough to explain or predict behavioural intention in certain user segments or for specific technologies.

*Research Question 3: Do perceived benefits mediate the relationship between UTAUT factors (represented by performance expectancy, effort expectancy, social influence, and facilitating conditions) and behavioural intention to use a mental health chatbot among professional employees?*

Hypothesis 3 (H32) that perceived benefits have a positive, mediating effect on the relationship between UTAUT and behavioural intention is not supported (path coefficient = 0.063, *p* = 0.263). The lack of support for Hypothesis 3 (H32) indicates that perceived benefits may not play a significant mediating role in the relationship between UTAUT factors and the behavioural intention to use mental health chatbots among professional employees. This finding contradicts the expectation based on UTAUT and TPB integration frameworks, where perceived benefits are hypothesised to mediate the relationship between UTAUT factors and behavioural intention. It suggests that the direct influence of UTAUT factors on behavioural intention might be stronger than previously thought, UTAUT factors such as performance expectancy, effort expectancy, social influence, and facilitating conditions are important, they may directly influence behavioural intention without the mediation of perceived benefits, or that other mediating factors could be at play in the context of mental health chatbots for the respondents. The diversity in professional fields, featuring engineers (34.2%), teachers (25.8%), and accountants (14.2%) as the most common occupations, strongly suggests that mental health chatbots are versatile enough to cater to a broad range of occupational backgrounds and diverse needs. This diversity further emphasises that the perceived benefits of these chatbots may not play a significant mediating role uniformly across professions, highlighting the complexity of factors influencing behavioural intentions in various occupational contexts. This finding urges a re-evaluation of the assumed mediating role of perceived benefits in technology adoption models.

*Research Question 4: Do attitudes towards chatbots moderate the relationship between UTAUT factors (represented by performance expectancy, effort expectancy, social influence, and facilitating conditions) and perceived benefits among professional employees?*

Hypothesis 4 (H4) that perceived attitudes towards chatbots positively strengthen the relationships between UTAUT and perceived benefits is not supported (path coefficient = 0.063, *p* = 0.094). This result diverges from the studies such as Grové (77), where a positive attitude was found to enhance the adoption of technology. The discrepancy between the current study and the prior literature might indicate that professional employees' attitudes towards mental health chatbots are influenced by factors other than just UTAUT factors, which might not have been adequately captured by the measurement instrument. For instance, some potential factors that might affect users’ attitudes towards chatbots are concerns about privacy, stigma, or the impersonal nature of chatbots.

Drawing connections to demographic data could further fortify this argument. For example, the impact of attitudes on the relationship between UTAUT factors and perceived benefits may differ among demographic segments. Young professionals within the dominant age group (25–34) (38.3%) might have a more positive attitude towards technology, thereby amplifying the impact of UTAUT factors on perceived benefits. On the contrary, individuals with more extensive work experience might bring a more cautious or sceptical attitude, potentially mitigating the influence of UTAUT factors. These factors might negatively influence the perceived usefulness, ease of use, social pressure, and availability of chatbots as mental health interventions and thus weaken the relationship between UTAUT factors and perceived benefits. This implies that attitudes towards technology are complex and multifaceted constructs that require a more comprehensive and nuanced approach to measure and understand them.

*Research Question 5: Do attitudes towards chatbots moderate the relationship between perceived benefits and behavioural intention to use a mental health chatbot among professional employees?*

Hypothesis 5 (H5) that perceived attitudes towards chatbots positively strengthen the relationships between perceived benefits and behavioural intention is also not supported (path coefficient = 0.183, *p* = 0.695). This finding contradicts the common belief that a positive attitude towards chatbots will always increase the likelihood of using them. This could imply that for the respondents, attitudes towards chatbots are not the only or the most important factor that affects their behavioural intention. Considering the prevalence of individuals with 1–3 years of work experience (38.3%), the study suggests that mental health chatbots may be especially relevant for new or young workers. This contradicts the notion that attitudes alone play a dominant role in shaping behavioural intentions, implying that factors such as trust, credibility, and personal relevance may exert a stronger influence on technology adoption decisions. Trust refers to the extent to which users believe that chatbots are reliable, secure, and accurate. Moreover, credibility refers to the extent to which users perceive that chatbots are competent, qualified, and authoritative. Personal relevance refers to the extent to which users feel that chatbots are relevant, useful, and suitable for their specific needs and preferences. These factors might moderate the relationship between perceived benefits and behavioural intention and thus explain the lack of support for the current research question. This result highlights the need to consider a broader range of factors that influence technology adoption in specific contexts, and not to rely solely on attitudes as the main predictor of behavioural intention.

*Removed factor loadings below 0.5*.

The initial confirmatory factor analysis (CFA) revealed that the factor loadings for EE1, EE3, EE4, EE5, and all FC loadings (FC1–FC5) were below the recommended threshold of 0.5. Moreover, the AVE for the UTAUT construct was also below the cut-off value of 0.5, indicating low convergent validity. These results suggested that some items of the UTAUT construct did not measure the same underlying concept and that the model fit was poor.

To improve the model fit and validity, the items with low factor loadings were removed, and the CFA was re-run. However, the new analysis showed that the model fit indices did not improve significantly and that the AVE for the UTAUT factors construct was 0.477 (still below 0.5). This indicated that removing the items did not solve the validity issue and that the UTAUT construct might be multidimensional rather than unidimensional.

Therefore, instead of discarding the items with low factor loadings, they were retained and justified based on theoretical and contextual grounds. The items of the EE and FC constructs were considered essential to capture the full breadth of the UTAUT construct, especially in the context of mental health chatbots for professional employees. It was argued that while some factor loadings were below the traditional cut-off of 0.5, they still provided valuable information and contributed to the overall variance explained by the UTAUT construct. Removing these items entirely could lead to a loss of important insights or oversimplify the complexity of the data. The limitations of the CFA were also acknowledged, and future studies were suggested to explore alternative models or methods to validate the UTAUT construct.

## Theoretical and practical implications

### Theoretical implications

The research findings provide valuable insights for both theoretical and practical applications. Theoretically, the study contributes to the existing literature on technology acceptance models by exploring the unique context of mental health chatbots for professional employees. The results challenge some of the established assumptions in the field, particularly regarding the role of perceived benefits and attitudes towards chatbots. This calls for a re-evaluation and potential refinement of the UTAUT and TPB frameworks in specific contexts, emphasising the need to consider a broader array of factors that influence technology adoption.

### Practical implications

From a practical standpoint, the findings have significant implications for developers, marketers, and policymakers involved in the design and promotion of mental health chatbots. Understanding that UTAUT factors significantly influence perceived benefits, but not necessarily behavioural intention, suggests that while it is crucial to ensure that mental health chatbots are easy to use, useful, socially accepted, and supported by necessary resources, these factors alone may not guarantee their adoption by professional employees. Developers need to focus on additional aspects such as privacy, accuracy, personal relevance, and trust to encourage usage.

The lack of a significant relationship between perceived benefits and behavioural intention indicates that professional employees might prioritise other considerations when deciding to use mental health chatbots. This highlights the importance of conducting in-depth user research to understand the specific needs and concerns of this demographic. For marketers, these insights can inform more targeted and effective marketing strategies that address specific barriers and motivations identified among professional employees.

For policymakers, the study underscores the importance of creating supportive environments that facilitate the adoption of mental health technologies. This includes not only ensuring the availability of the necessary infrastructure but also addressing broader issues such as privacy concerns, stigma associated with mental health, and the credibility of digital mental health solutions.

In conclusion, this study provides a nuanced understanding of the factors influencing the adoption of mental health chatbots by professional employees. It challenges some prevailing assumptions in the field of technology acceptance and highlights the need for a more comprehensive approach to understanding and addressing the factors that influence technology adoption, particularly in the context of mental health.

## Limitations and future studies

### Limitations

This research, similar to any empirical study, faces several limitations that need to be acknowledged. Firstly, the study's reliance on convenience sampling limits the generalisability of the findings. Since participants were selected based on accessibility rather than through a random process, the sample may not accurately represent the entire population of professional employees in Malaysia. Consequently, the results may be biased towards the characteristics and preferences of the particular group that was more readily available for the study. Another limitation is the cross-sectional nature of the research design. Data were collected at a single point in time, which restricts the ability to conclude the causal relationships between variables. Longitudinal studies would be more effective in understanding how attitudes and perceptions towards mental health chatbots evolve over time and how these changes impact behavioural intentions.

The study also relied solely on self-reported measures, which can be subject to biases such as social desirability or response bias. Participants may provide answers that they believe are expected or acceptable rather than their true feelings or intentions. This may lead to inaccuracies in measuring the constructs of interest. Additionally, the research focused on a specific application of technology—mental health chatbots—within a particular professional context. This specific focus might limit the applicability of the findings to other types of technology or user populations.

### Future studies

Future research can address these limitations and explore additional areas to deepen the understanding of technology adoption in the context of mental health. Studies can employ random sampling techniques to enhance the representativeness and generalisability of the findings. Longitudinal research designs could be utilised to examine changes in attitudes and behavioural intentions over time, providing more dynamic insights into the factors influencing technology adoption.

Further studies might also explore the role of other potential moderating and mediating variables, such as personal innovativeness, technology anxiety, or perceived risk, which could influence the relationship between UTAUT factors, perceived benefits, and behavioural intention. Investigating actual usage behaviour, rather than just behavioural intentions, could provide more concrete evidence of the factors influencing the adoption and sustained use of mental health chatbots. Moreover, qualitative methods such as interviews or focus groups could be employed to gain deeper insights into the reasons behind users' perceptions and intentions. Expanding the research to different contexts, technologies, and user groups can also enhance the understanding of the applicability and robustness of the UTAUT and TPB models in various settings. For instance, examining the adoption of mental health chatbots in different cultural contexts or within different professional sectors could reveal valuable cross-cultural and industry-specific insight.

## Conclusion

This study explored the factors influencing professional employees' behavioural intention to use mental health chatbots in Malaysia by integrating the Unified Theory of Acceptance and Use of Technology (UTAUT) and the Theory of Planned Behaviour (TPB). The research offers a comprehensive understanding of technology adoption in the mental health domain, particularly within a professional workforce context.

The findings revealed that UTAUT constructs performance expectancy, effort expectancy, social influence, and facilitating conditions significantly shape users' perceptions of the benefits of mental health chatbots. When these technologies are seen as useful, easy to navigate, socially supported, and technically accessible, professionals are more likely to recognise their value. However, perceived benefits did not significantly influence behavioural intention, challenging the assumption that perceived usefulness alone drives adoption. This suggests that other psychological or contextual factors may play a more decisive role in shaping professionals' willingness to engage with mental health chatbots.

Furthermore, the study found limited evidence supporting the moderating role of attitudes towards chatbots. While attitudes are traditionally considered influential in technology acceptance models, their impact in this context was weak. This points to the importance of trust, credibility, and relevance over general sentiment when it comes to mental health technologies.

From a theoretical perspective, the study contributes to the refinement of UTAUT and TPB by highlighting the limitations of perceived benefits and attitudes as predictors in professional mental health settings. Practically, the findings offer actionable insights for developers, policymakers, and mental health practitioners. Developers should focus on enhancing chatbot trustworthiness, personalisation, and ethical safeguards. Policymakers should address barriers such as stigma, digital literacy gaps, and unequal access to mental health technologies.

To strengthen future research, longitudinal studies are recommended to capture evolving user perceptions and behaviours over time. Additionally, incorporating qualitative methods such as interviews or focus groups could provide deeper insights into user experiences and adoption barriers. Expanding the scope to include diverse professional sectors and cultural contexts would also improve generalisability and relevance.

In summary, while mental health chatbots present promising opportunities for supporting professionals in managing psychological well-being, their adoption is influenced by a complex interplay of technological, psychological, and contextual factors. Addressing these intricacies is essential for designing inclusive, effective, and sustainable digital mental health solutions.

## Data Availability

The raw data supporting the conclusions of this article will be made available by the authors, without undue reservation.

## Appendix A Table for survey items.

**Table:**

Construct	Item no.	Item description	Source
Performance expectancy	PE1	“A mental health chatbot would be useful for mental health needs.”	(22, 101)
	PE2	“Using a mental health chatbot would enable access to mental health services anytime and anywhere.”	
	PE3	“Using a mental health chatbot would enhance mental health outcomes.”	
	PE4	“Using a mental health chatbot would increase mental health awareness.”	
	PE5	“Using a mental health chatbot would reduce the need for professional help or medication.”	
Effort expectancy	EE1	“Learning to use a mental health chatbot would be easy.”	(101)
	EE2	“Interaction with a mental health chatbot would be clear and understandable.”	
	EE3	“A mental health chatbot would be easy to use.”	
	EE4	“A mental health chatbot would have a simple and user-friendly interface.”	
	EE5	“A mental health chatbot would respond quickly and accurately to queries.”	
Social influence	SI1	“People who influence behaviour think that using a mental health chatbot is beneficial.”	(88)
	SI2	“People who are important think that using a mental health chatbot is advisable.”	
	SI3	“Other people use a mental health chatbot successfully.”	
	SI4	“Using a mental health chatbot is socially acceptable and desirable.”	
	SI5	“Using a mental health chatbot would increase connection and support from others.”	
Facilitating conditions	FC1	“The resources necessary to use a mental health chatbot are available, such as a smartphone or computer and an internet connection.”	(101)
	FC2	“The knowledge necessary to use a mental health chatbot is sufficient, such as how to install, operate, and troubleshoot it.”	
	FC3	“A mental health chatbot is compatible with other technologies in use, such as messaging apps or voice assistants.”	
	FC4	“There is sufficient guidance and support available for using a mental health chatbot, such as tutorials, FAQs, or customer service.”	
	FC5	“There are no technical or legal barriers that prevent using a mental health chatbot, such as bugs, errors, or privacy violations.”	
Perceived benefits	PB1	“Using a mental health chatbot would save time and money compared with other forms of mental healthcare.”	(88)
	PB2	“Using a mental health chatbot would provide personalised and confidential support that matches needs and preferences.”	
	PB3	“Using a mental health chatbot would help cope with stress and improve well-being by providing information, feedback, and strategies.”	
	PB4	“Using a mental health chatbot would increase self-efficacy and empowerment in managing one's own mental health.”	
	PB5	“Using a mental health chatbot would enhance communication and social skills by providing practice and feedback.”	
Attitude towards chatbots	AC1	“The idea of using a chatbot for mental health is appealing.”	(77)
	AC2	“Talking to a chatbot about mental health issues is comfortable.”	
	AC3	“A chatbot can provide accurate and helpful information.”	
	AC4	“Interacting with a chatbot for mental health is enjoyable.”	
	AC5	“The chatbot is an intelligent and reliable agent.”	
Behavioural intention	BI1	“Using a mental health chatbot in the future is intended.”	(101)
	BI2	“Using a mental health chatbot frequently is planned.”	
	BI3	“Using a mental health chatbot regularly is predicted.”	
	BI4	“Using a mental health chatbot is recommended to others who need it.”	
	BI5	“Trying different features and functions of a mental health chatbot is willing.”	
